# Global burden of disease for gastric cancer from 1990 to 2023 and projections of the burden to 2035

**DOI:** 10.1097/MD.0000000000050643

**Published:** 2026-09-11

**Authors:** Liying Ma, Juan Ou, Zijun Qiu, Xi Lin, Dingyu Guo, Bing Yan

**Affiliations:** a The Fourth Clinical Medical College, Guangzhou University of Chinese Medicine, Shenzhen, China.

**Keywords:** BAPC modeling, gastric cancer, global burden of disease, health inequality, socio-demographic index

## Abstract

Gastric cancer (GC) remains a major global health challenge, contributing substantially to cancer incidence and mortality worldwide. This study aimed to evaluate the worldwide disease burden and evolving trends of GC utilizing the Global Burden of Disease 2023 database, intending to offer a robust scientific foundation for public health policy. Utilizing pertinent information from the Global Burden of Disease 2023 database, we calculated the estimated annual percentage change to measure temporal trends. We applied age-period-cohort models to analyze the impacts of time periods and cohorts from 1990 to 2023. Additionally, we explored the correlation between the burden of GC and the socio-demographic index through frontier analysis and health inequality evaluation while forecasting future trends utilizing Bayesian age-period-cohort models. From 1990 to 2023, the global cases of GC rose by 20.04%, reaching 1262,000; the total number of deaths increased from 912,483 to 934,795; however, cancer-related disability-adjusted life years (DALYs) declined by 8.85% compared to 1990. The age-standardized incidence rate, age-standardized mortality rate, and age-standardized DALY rate of GC have generally demonstrated a downward trend since 1990. The disease burden is notably greater in males than in females. Bayesian age-period-cohort projections indicate that by 2035, GC incidence is anticipated to reach 1,159,745, while the expected numbers of deaths and DALYs are forecasted to decline to 667,145 and 15,783,072, respectively; additionally, the impact of GC in older age demographics is projected to increase significantly. Although the age-standardized incidence, mortality, and disability rates of GC have declined globally from 1990 to 2023, the absolute number of incident cases, deaths, and DALYs remains substantial, indicating a persistent global health burden. Future prevention strategies should consider differences in demographic structure, socioeconomic development, and regional disease patterns.

## 1. Introduction

Gastric cancer (GC) ranks among the predominant malignant conditions globally, imposing a significant burden on society and affected individuals.^[1,2]^ According to data from the International Agency for Research on Cancer, in 2018, GC was identified as the fifth most prevalent malignant tumor internationally, contributing to 8.2% of all fatalities associated with cancer.^[3]^ Despite a declining trend in the incidence and mortality rates of GC over the past few decades, projections indicated that approximately 1089,000 new cases and 769,000 deaths occurred worldwide in 2020. The medical challenges stemming from GC remain considerable and cannot be overlooked.^[4]^

Numerous studies have investigated the pathogenesis and treatment approaches for GC; however, macro-epidemiologic analyses derived from the Global Burden of Disease (GBD) database still present some deficiencies. The existing literature predominantly emphasizes single countries or regions, failing to address the spatial and temporal variations on a global level, and the relationship between demographic shifts and the variation in risk factors remains insufficiently measured.^[5,6]^ Consequently, examining the burden of disease and trends along with gender differences and specific age subcategories is crucial for formulating effective health policies and identifying potential interventions.

Previous GBD-based studies have provided important estimates of GC burden; however, many analyses focused primarily on descriptive epidemiological trends. The present study extends previous work by using updated GBD 2023 estimates and integrating multiple analytical approaches, including inequality assessment, frontier analysis, age-period-cohort (APC) modeling, and Bayesian age-period-cohort (BAPC) forecasting. This framework enables comprehensive evaluation of historical changes, socioeconomic disparities, and future burden trajectories.^[7,8]^

This study aims to conduct a comprehensive examination of the worldwide occurrence of GC from 1990 to 2023, utilizing the GBD database. By investigating the trends in critical indicators of GC: including incidence, mortality, and disability-adjusted life years (DALY): the study evaluates the correlation between these variations and the socio-demographic index (SDI). The analysis presented herein can illuminate the characteristics of the global distribution of GC as well as the socioeconomic factors contributing to it, thereby laying the groundwork for the formulation of effective preventive and treatment strategies. Ultimately, this research seeks to enhance the health outcomes and quality of life for individuals afflicted with GC. The study objectives were refined into 3 aims: characterize global and regional GC burden trends; evaluate socioeconomic inequalities and possible related factors; and predict future burden trajectories using BAPC models.

## 2. Method and material

### 2.1. Data sources

This study used estimates from the GBD 2023 study, which is coordinated by the Institute for Health Metrics and Evaluation. The data were extracted from the GBD Results Tool (https://ghdx.healthdata.org/gbd-results-tool). The GBD database provides standardized estimates of disease burden, including incidence, mortality, prevalence, DALYs, and corresponding uncertainty intervals (UIs). The 95% UIs were obtained directly from the GBD 2023 database. In the GBD framework, UIs are generated by sampling from the posterior distribution of the estimation models, incorporating uncertainty from input data, model parameters, and other estimation processes. The 95% UI represents the 2.5th and 97.5th percentiles of the posterior distribution.

GC was defined according to the International Classification of Diseases (ICD) coding system used in the GBD framework, including ICD-9 code 151.9 and ICD-10 code C16.9. All GC categories included in the GBD definition were considered, and no specific histological subtype was excluded.

The analysis included estimates stratified by sex (male, female, and both sexes), age groups (all ages and specific 5-year age groups where applicable), and geographical locations classified according to the GBD hierarchical regional framework, including global, super-regional, regional, and national levels. SDI quintiles were also incorporated to evaluate associations between socioeconomic development and GC burden.^[9,10]^

### 2.2. Ethical considerations

This research utilized data that is publicly available from GBD 2023. The dataset comprises de-identified information that is not directly linked to any individuals, thus negating the necessity for additional ethical review or informed consent.

### 2.3. Study cohort

Data from the global population affected by GC was analyzed during the period from 1990 to 2023 for this study. The diagnosis of GC was based on the International Classification of Diseases-9 (ICD-9) and ICD-10 codes, specifically ICD-9: 151.9 and ICD-10: C16.9.^[11]^ Data were obtained from the GBD 2023 database. GC was defined according to the corresponding ICD diagnostic categories used in the GBD framework. Estimates were analyzed by sex, age group, and geographical region. UIs were obtained from the GBD estimation framework. For temporal trends, the estimated annual percentage change (EAPC) was calculated using a log-linear regression model:

ln(Y)=α+βX; EAPC = 100 × (exp(β)-1).

APC models were applied to evaluate independent effects associated with age, period, and cohort. BAPC models were used for future projections under the assumption that historical trends would continue.

### 2.4. SDI and epidemiological indicators

The SDI serves as a composite measure reflecting the underlying social and economic conditions. It is derived from indicators such as the total fertility rate among females under 25 years, the average years of education for females aged 15 and older, and the Lagged Distribution Index of Per Capita Income.^[12,13]^ The SDI ranges from 0 to 1, where a higher SDI signifies a more favorable socioeconomic status. This study concentrated on the selection of epidemiological indicators, particularly focusing on (Age-Standardized Incidence Rate [ASIR]/100,000/year), age-standardized mortality rates (ASMR/100,000/year), and age-standardized disability-adjusted life-year rates (age-standardized DALY rate [ASDR]/100,000/year).

### 2.5. Statistical analysis

Statistical analyses were performed to comprehensively evaluate the temporal trends, contributing factors, socioeconomic inequalities, and future trajectories of GC burden. First, temporal trends in GC incidence, mortality, DALYs, and corresponding age-standardized rates were assessed using the EAPC and average annual percentage change (AAPC). The EAPC was calculated using a log-linear regression model: ln(Y)=α+βX+ϵ, where Y represents the age-standardized rate, X represents the calendar year, α represents the intercept, β represents the regression coefficient, and ε represents the error term. The EAPC was calculated as EAPC = 100 × (eβ − 1), with values greater than zero indicating an increasing trend and values less than zero indicating a decreasing trend. Compared with EAPC, which describes the overall long-term trend during the entire study period, AAPC summarizes average annual changes over specific time intervals and is particularly useful when temporal trends vary across different periods.

Decomposition analysis was performed to quantify the relative contributions of population growth, population aging, and epidemiological changes to variations in the absolute number of GC cases, deaths, and DALYs between 1990 and 2023. This analysis allowed differentiation between demographic-driven changes and changes associated with disease risk patterns.

To evaluate socioeconomic disparities, the association between GC burden and the SDI was assessed. Inequality analysis was conducted to characterize differences in disease burden across socioeconomic development levels, while frontier analysis was applied to estimate the minimum achievable burden according to SDI levels and identify potential opportunities for improvement. A greater distance from the frontier indicated greater potential for reducing GC burden under comparable socioeconomic conditions.

APC models were applied to evaluate the independent effects of age, period, and birth cohort on GC burden. The APC model can be expressed as log(Ya,p)=μ+αa+πp+γc+ϵ, where Ya,p represents the estimated burden for age group a during period p, α represents age effects, π represents period effects, and γ represents cohort effects. Because age, period, and cohort variables are linearly dependent (cohort = period − agecohort = period-agecohort = period − age), APC models face an inherent identification problem. In this study, constrained estimation approaches were applied to address this issue and allow interpretation of relative age, period, and cohort effects.

Finally, BAPC models were used to project GC incidence, mortality, and DALY trends from 2024 to 2035. The BAPC model integrates APC structures with Bayesian inference and estimates future disease burden based on historical age, period, and cohort patterns. These projections were interpreted under the assumption that historical trends would continue, and therefore represent expected future trajectories rather than definitive predictions. Overall, the combination of these analytical approaches enabled a comprehensive assessment of historical changes, socioeconomic inequalities, demographic influences, and future burden patterns of GC.

## 3. Results

### 3.1. GBD for GC, 1990–2023

In 2023, the global burden associated with GC is considerable (Table 1 and S1, Supplemental Digital Content 1). Worldwide, the estimated incidences of GC reached approximately 1262,000, reflecting a 20.04% rise since 1990 and indicating an overall upward trajectory. The ASIR declined from 26.32 (95% UI:23.14,29.51) per 100,000 individuals in 1990 to 13.81 (95% UI: 11.69, 16.63) in 2023, showcasing an EAPC of −2.20 (95% concentration index [CI]: −2.29, −2.10). Regarding mortality, fatalities attributed to GC rose by 2.45%, increasing from 912,483 to 934,795. The (ASMR) fell from 23.34 (95% UI: 20.64, 26.45) per 100,000 to 10.27 (95% UI: 8.75, 11.67) per 100,000, with an EAPC of −2.77 (95% CI: −2.88, −2.65). The ASDR decreased from 592.53 (95% UI: 518.90, 672.04) to 246.70 (95% UI: 211.92, 285.49) per 100,000 population, with an EAPC of −2.98 (95% CI: −3.11, −2.85). (Table 1)

**Table 1:** The ASIR, ASMR and ASDR of GC in 1990 and 2023 by SDI quintiles and GBD regions, with EAPC and PC from 1990 to 2023.

Characteristics	Age-standardized incidence rate per 100,000 population				Age-standardized mortality rate per 100,000 population				Age-standardized DALY rate per 100,000 population			
	1990 no (95% UI)	2023 no (95% UI)	EAPC no (95% CI)	PC no (95% CI)	1990 no (95% UI)	2023 no (95% UI)	EAPC no (95% CI)	PC no (95% CI)	1990 no (95% UI)	2023 no (95% UI)	EAPC no (95% CI)	PC no (95% CI)
Global	26.32 (23.14,29.51)	13.81 (11.69,16.63)	−2.20 (−2.29, −2.10)	−47.51 (−55.31, −38.41)	23.34 (20.64,26.45)	10.27 (8.75,11.67)	−2.77 (−2.88, −2.65)	−59.48 (−67.25, −51.43)	592.53 (518.90,672.04)	246.70 (211.92,285.49)	−2.98 (−3.11, −2.85)	−61.07 (−68.95, −53.01)
Sex												
Male	35.90 (30.90,42.56)	19.89 (15.68,24.20)	−1.98 (−2.08, −1.88)	−44.61 (−54.68, −32.66)	31.72 (27.07,38.10)	14.49 (11.34,17.26)	−2.60 (−2.71, −2.48)	−54.33 (−62.40, −43.44)	797.47 (670.32,962.39)	342.85 (268.44,417.01)	−2.83 (−2.96, −2.70)	−57.01 (−64.91, −45.15)
Female	18.18 (15.99,20.76)	8.50 (6.93,10.07)	−2.64 (−2.75, −2.54)	−53.26 (−62.14, −44.41)	16.39 (14.21,18.91)	6.64 (5.52,7.99)	−3.11 (−3.24, −2.98)	−59.48 (−67.25, −51.43)	410.21 (354.17,475.26)	159.67 (133.67,193.46)	−3.27 (−3.41, −3.12)	−61.07 (−68.95, −53.01)
SDI quintile												
High	30.29 (27.53,33.04)	15.65 (13.42,18.57)	−2.24 (−2.35, −2.13)	−48.32 (−54.79, −39.32)	25.39 (23.13,27.86)	10.27 (8.87,11.86)	−3.03 (−3.17, −2.90)	−63.57 (−68.86, −58.83)	644.09 (579.68,708.89)	236.78 (211.02,275.85)	−3.36 (−3.52, −3.21)	−67.38 (−71.75, −63.03)
High middle	37.09 (30.88,46.21)	15.99 (13.16,20.40)	−2.84 (−2.97, −2.71)	−56.89 (−64.76, −45.41)	35.45 (29.53,44.43)	11.88 (10.03,14.30)	−3.62 (−3.78, −3.46)	−71.98 (−79.05, −64.81)	906.68 (759.94,1128.89)	282.26 (240.94,338.49)	−3.88 (−4.04, −3.71)	−74.53 (−80.82, −68.57)
Middle	16.03 (13.08,20.76)	10.36 (8.57,13.51)	−1.55 (−1.65, −1.45)	−35.36 (−49.48, −20.50)	15.63 (12.89,19.95)	9.13 (7.44,11.43)	−1.87 (−1.98, −1.75)	−46.67 (−61.21, −27.39)	410.66 (338.65,518.22)	231.88 (188.82,291.27)	−1.99 (−2.11, −1.87)	−48.48 (−62.20, −28.81)
Low middle	10.20 (7.91,13.10)	7.52 (5.75,9.25)	−0.95 (−1.03, −0.87)	−26.32 (−47.04,1.60)	10.12 (7.83,12.68)	7.63 (5.88,9.34)	−0.88 (−0.96, −0.79)	−25.48 (−53.74,10.33)	265.83 (204.01,337.95)	195.66 (150.07,239.14)	−0.98 (−1.07, −0.90)	−27.15 (−54.40,7.56)
Low	10.40 (7.40,14.17)	8.97 (6.10,11.92)	−0.66 (−0.77, −0.55)	−13.70 (−43.47,27.24)	10.09 (6.94,13.64)	9.21 (6.39,12.41)	−0.47 (−0.59, −0.35)	−9.30 (−48.06,60.74)	280.23 (193.99,381.77)	249.74 (175.97,336.35)	−0.59 (−0.74, −0.44)	−10.80 (−48.15,52.56)
GBD region												
Andean Latin America	25.17 (21.87,28.71)	19.00 (16.27,22.44)	−1.19 (−1.52, −0.87)	−24.53 (−38.24, −8.44)	25.61 (22.31,29.30)	17.99 (15.46,20.84)	−1.37 (−1.71, −1.02)	−33.36 (−48.30, −17.35)	602.19 (522.87,687.48)	416.17 (358.77,481.68)	−1.38 (−1.71, −1.04)	−32.89 (−47.92, −16.39)
Australasia	10.85 (9.94,11.77)	6.60 (5.72,7.50)	−1.54 (−1.61, −1.47)	−39.23 (−47.66, −29.17)	8.07 (7.67,8.47)	3.71 (3.24,4.09)	−2.29 (−2.34, −2.24)	−55.10 (−61.80, −48.54)	177.76 (169.33,187.87)	80.39 (72.31,88.46)	−2.35 (−2.40, −2.30)	−56.43 (−62.55, −50.02)
Caribbean	12.29 (11.16,13.40)	8.40 (7.31,9.65)	−1.18 (−1.35, −1.01)	−31.65 (−38.03, −23.60)	11.84 (10.77,12.86)	7.88 (6.74,8.87)	−1.30 (−1.51, −1.10)	−31.00 (−41.05, −20.07)	287.53 (258.89,318.98)	199.55 (168.33,228.71)	−1.18 (−1.42, −0.94)	−27.89 (−40.49, −13.19)
Central Asia	27.26 (25.39,28.62)	11.91 (10.97,13.35)	−2.30 (−2.44, −2.17)	−56.32 (−59.87, −50.95)	26.96 (25.10,28.35)	11.58 (10.65,12.80)	−2.34 (−2.47, −2.20)	−62.72 (−66.38, −58.97)	731.87 (680.86,771.42)	299.11 (276.53,333.43)	−2.61 (−2.72, −2.50)	−63.85 (−67.22, −59.91)
Central Europe	18.01 (17.18,18.67)	8.03 (7.48,8.62)	−2.46 (−2.56, −2.36)	−55.40 (−57.98, −52.17)	18.43 (17.65,19.09)	7.61 (7.09,8.12)	−2.70 (−2.80, −2.61)	−61.71 (−64.06, −59.61)	442.55 (426.04,457.65)	179.56 (167.30,192.07)	−2.76 (−2.86, −2.67)	−61.48 (−63.98, −59.32)
Central Latin America	21.54 (20.38,22.94)	12.67 (11.65,13.66)	−1.73 (−1.81, −1.65)	−41.19 (−46.44, −35.87)	21.38 (20.19,22.80)	10.89 (10.18,11.46)	−2.19 (−2.28, −2.10)	−50.96 (−55.36, −46.10)	492.81 (468.65,522.95)	263.94 (250.28,278.01)	−2.00 (−2.10, −1.89)	−47.80 (−52.26, −42.68)
Central sub−Saharan Africa	8.32 (5.40,11.62)	7.95 (4.95,10.16)	−0.21 (−0.36, −0.07)	−4.35 (−33.24,36.17)	8.47 (5.50,11.43)	8.06 (5.00,10.64)	−0.28 (−0.42, −0.14)	1.58 (−40.35,59.04)	223.18 (150.59,298.45)	213.80 (136.18,275.30)	−0.23 (−0.40, −0.06)	3.22 (−39.76,60.50)
East Asia	53.12 (43.09,65.30)	24.54 (19.52,31.77)	−2.72 (−2.92, −2.52)	−53.79 (−62.82, −40.05)	50.07 (40.56,62.01)	16.46 (13.75,20.70)	−3.81 (−4.07, −3.55)	−73.44 (−80.53, −63.83)	1290.00 (1051.46,1585.33)	387.16 (319.38,490.26)	−4.13 (−4.39, −3.86)	−76.97 (−82.81, −69.82)
Eastern Europe	30.22 (28.44,32.15)	13.66 (12.57,14.95)	−2.46 (−2.53, −2.39)	−54.79 (−58.81, −49.81)	27.88 (26.41,29.45)	10.91 (10.18,11.53)	−2.94 (−3.03, −2.85)	−64.72 (−69.16, −60.29)	749.51 (709.29,792.20)	275.50 (259.11,290.75)	−3.24 (−3.35, −3.13)	−66.02 (−70.10, −61.64)
Eastern sub-Saharan Africa	9.41 (6.79,13.41)	8.46 (5.64,10.97)	−0.46 (−0.59, −0.33)	−10.09 (−41.12,35.91)	8.68 (6.10,12.06)	8.44 (5.65,10.94)	−0.23 (−0.34, −0.11)	−3.74 (−46.15,70.62)	272.36 (195.15,381.03)	251.51 (168.56,327.37)	−0.47 (−0.64, −0.29)	−8.39 (−49.46,60.63)
High-income Asia Pacific	65.03 (60.18,68.98)	25.94 (22.73,31.25)	−3.01 (−3.10, −2.92)	−60.12 (−65.31, −50.15)	38.15 (35.13,40.28)	13.13 (11.16,15.66)	−3.52 (−3.60, −3.44)	−67.39 (−73.99, −58.98)	933.81 (840.93,984.75)	267.68 (237.06,326.30)	−4.07 (−4.16, −3.98)	−72.76 (−77.61, −65.18)
High-income North America	8.48 (7.53,9.64)	5.27 (4.50,6.02)	−1.61 (−1.69, −1.53)	−37.82 (−48.10, −24.35)	5.74 (5.35,6.24)	2.93 (2.62,3.22)	−2.15 (−2.24, −2.06)	−44.31 (−52.83, −34.91)	132.62 (123.76,142.87)	71.80 (64.91,79.20)	−1.97 (−2.08, −1.86)	−38.02 (−47.46, −27.27)
North Africa and Middle East	16.30 (10.94,21.42)	10.61 (8.04,12.80)	−1.33 (−1.43, −1.23)	−34.91 (−52.75, −6.45)	16.43 (10.98,21.53)	9.79 (7.53,11.83)	−1.60 (−1.68, −1.51)	−41.48 (−64.55, −3.84)	396.27 (265.79,514.42)	219.55 (164.34,264.65)	−1.84 (−1.93, −1.75)	−44.78 (−65.96, −13.74)
Oceania	20.69 (12.66,32.77)	18.08 (10.47,27.57)	−0.68 (−0.79, −0.57)	−12.61 (−42.20,28.09)	20.33 (12.42,31.18)	18.40 (10.93,28.06)	−0.60 (−0.71, −0.49)	4.96 (−37.66,64.75)	514.64 (304.33,794.37)	472.87 (283.26,722.54)	−0.51 (−0.63, −0.39)	7.97 (−36.68,70.80)
South Asia	8.72 (6.33,11.82)	6.69 (4.61,8.77)	−0.93 (−1.06, −0.80)	−23.32 (−49.57,14.79)	8.62 (6.25,11.80)	7.03 (4.85,9.44)	−0.72 (−0.86, −0.57)	−19.89 (−57.20,43.18)	233.61 (171.29,318.06)	185.74 (130.32,249.82)	−0.85 (−0.99, −0.70)	−22.44 (−57.85,36.79)
Southeast Asia	10.18 (7.66,13.21)	7.67 (5.90,10.19)	−0.98 (−1.08, −0.89)	−24.65 (−43.62,1.86)	9.95 (7.49,12.74)	7.03 (5.62,9.08)	−1.19 (−1.29, −1.10)	−33.41 (−54.61, −1.95)	264.21 (197.31,336.96)	193.37 (151.24,250.82)	−1.07 (−1.17, −0.97)	−31.79 (−54.70,3.87)
Southern Latin America	19.86 (18.94,21.08)	11.62 (10.61,12.55)	−1.61 (−1.69, −1.53)	−41.48 (−47.25, −35.55)	19.88 (19.01,21.05)	10.28 (9.51,10.88)	−1.95 (−2.03, −1.87)	−49.63 (−55.51, −43.93)	457.39 (436.29,482.30)	234.77 (220.37,248.57)	−1.95 (−2.04, −1.86)	−48.69 (−55.14, −42.62)
Southern sub-Saharan Africa	8.60 (6.52,11.10)	6.19 (4.92,7.55)	−1.27 (−1.50, −1.04)	−27.98 (−44.61, −3.15)	8.51 (6.52,10.93)	5.33 (4.23,6.62)	−1.65 (−1.87, −1.42)	−32.24 (−55.34,4.17)	214.71 (163.04,279.83)	137.87 (108.29,174.51)	−1.53 (−1.75, −1.30)	−29.76 (−53.92,11.00)
Tropical Latin America	18.59 (17.17,20.27)	9.42 (8.68,10.01)	−2.04 (−2.09, −1.98)	−49.31 (−53.44, −44.68)	18.80 (17.39,20.58)	8.69 (8.09,9.19)	−2.26 (−2.31, −2.21)	−53.98 (−58.41, −48.48)	445.64 (412.77,484.90)	210.30 (199.04,220.95)	−2.21 (−2.27, −2.16)	−50.12 (−54.59, −44.13)
Western Europe	17.56 (16.32,18.95)	8.78 (7.62,9.89)	−2.15 (−2.19, −2.12)	−50.01 (−56.35, −42.55)	15.31 (14.57,16.08)	5.62 (5.02,6.14)	−3.13 (−3.22, −3.03)	−64.99 (−68.63, −60.73)	331.42 (319.42,347.14)	121.89 (111.27,132.98)	−3.10 (−3.19, −3.01)	−64.21 (−68.26, −59.81)
Western sub-Saharan Africa	7.29 (5.28,9.69)	5.50 (3.96,6.88)	−1.02 (−1.20, −0.84)	−24.57 (−48.04,8.60)	7.38 (5.30,9.64)	5.64 (4.01,7.07)	−1.00 (−1.17, −0.83)	−26.91 (−54.85,16.01)	199.31 (145.08,260.47)	150.72 (108.32,187.20)	−1.03 (−1.24, −0.82)	−27.07 (−55.57,14.15)

**Figure 1.**
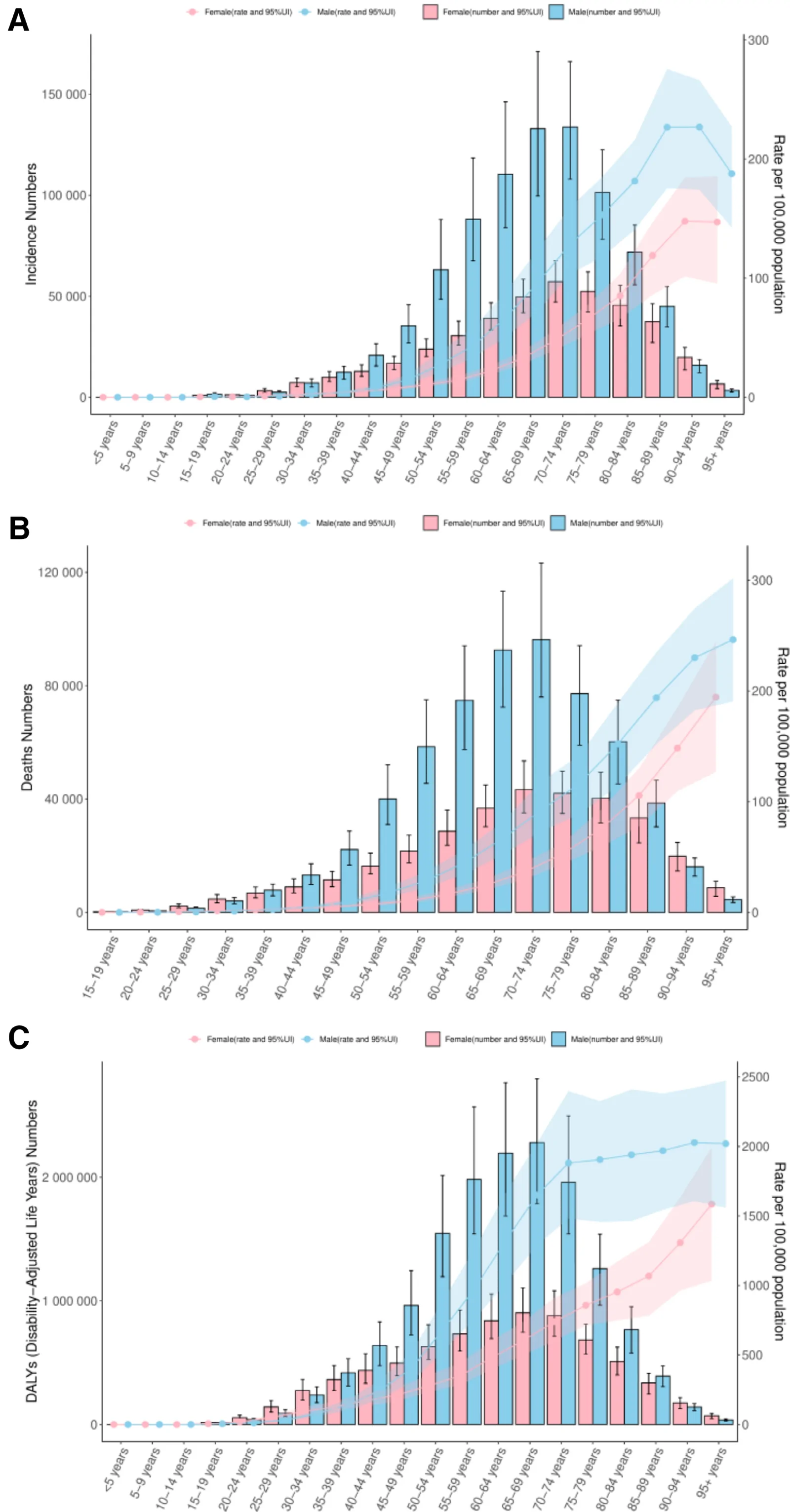
Cohort analysis. (A) number of incidences; (B) number of deaths; (C) number of DALYs. DALY = disability-adjusted life year.

In terms of gender, males have a higher burden of GC than females.The number of incidences, deaths, DALYs, ASIR, ASMR, and ASDR were higher in males than in females globally in 2023.Both ASIR, ASMR, ASDR showed an overall increasing trend with age, and the number of incidences, deaths, and DALYs first increased and then decreased. (Fig. 1)

**Figure 2.**
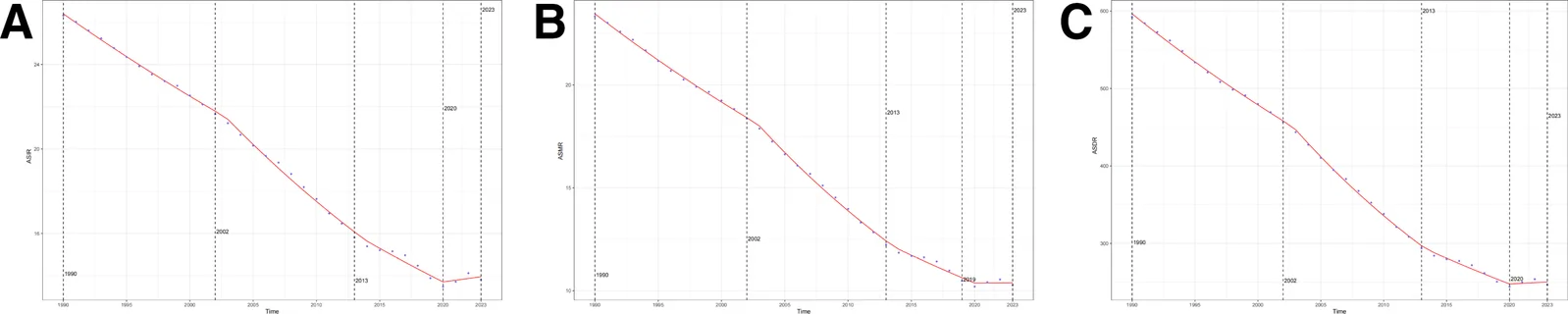
AAPC analysis. (A) ASIR; (B) ASMR; (C) ASDR. AAPC = average annual percentage change, ASIR = age-standardized incidence rate, ASMR = age-standardized mortality rate, ASDR = age-standardized DALY rate.

From the AAPC analysis (Figure 2, Table S2, Supplemental Digital Content 2), the ASIR of GC showed a broadly decreasing trend since 1990 (AAPC = −1.909; 95% CI: −2.086 to −1.732),and the most significant decrease was observed from 2002 to 2013 (AAPC = −2.822 (−3.015 to −2.629), *P* < .05). ASIR for both males and females had similar trends and magnitude of change as the overall population. ASMR and ASDR showed similar trends.

### 3.2. Regional burden trends in GC

**Figure 3.**
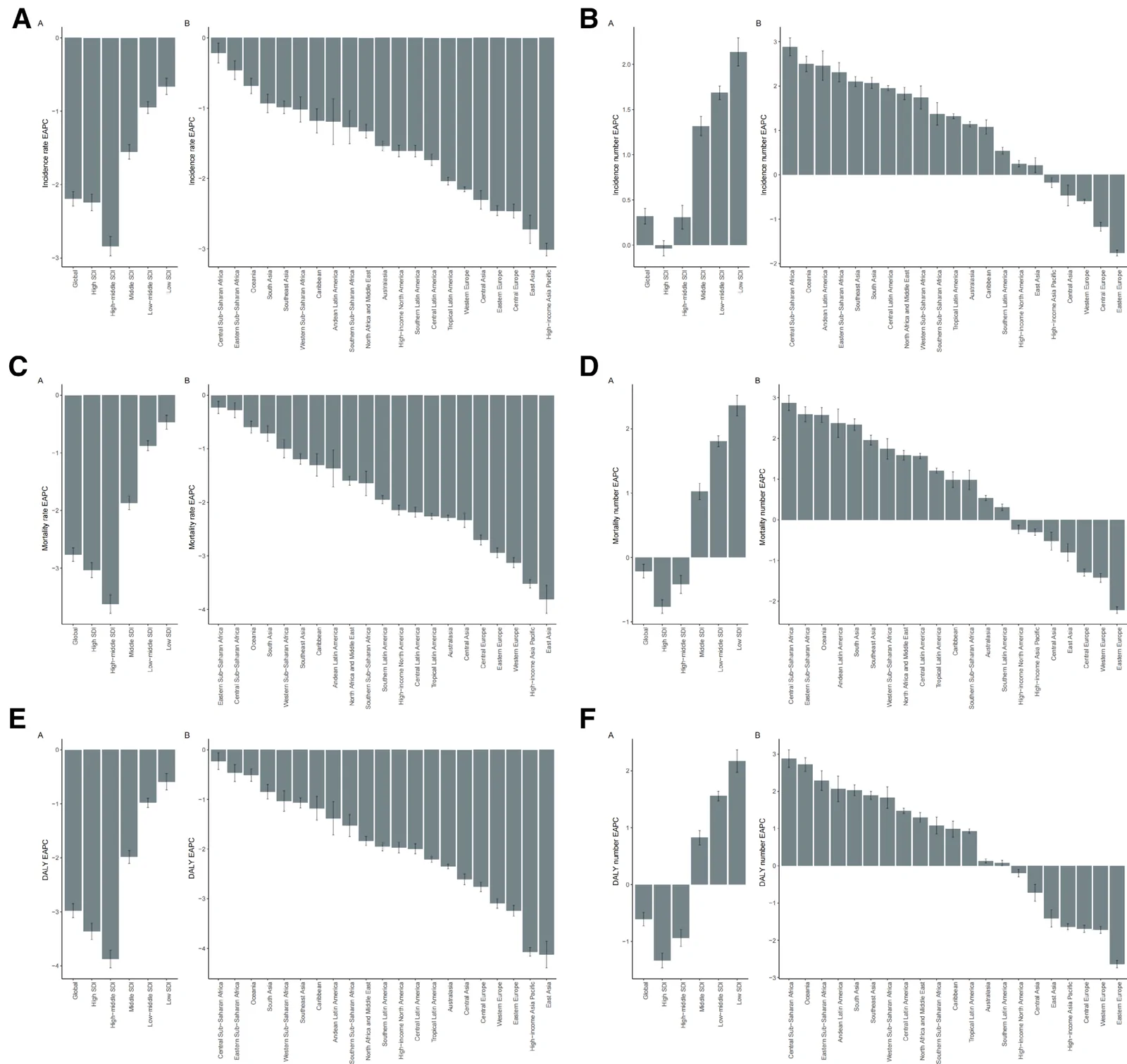
EAPC analysis. (A, B) ASIR and number of incidences; (C, D) ASMR and number of deaths; (E, F) ASDR and number of DALYs. AAPC = average annual percentage change, ASIR = age-standardized incidence rate, ASMR = age-standardized mortality rate, ASDR = age-standardized DALY rate, DALY = disability-adjusted life year.

At the regional level, (Figure 3, Table 1) in 2023, ASIR was highest in the High-income Asia Pacific region (25.94 per 100,000) and lowest in the High-income North America region (5.27 per 100,000). The East Asia region has the highest incidence (582,995) and the Oceania region has the lowest (1516). ASIRs all showed a decreasing trend, with the largest decrease in the High-income Asia Pacific region (EAPC = −3.01,95% CI: −3.10,-2.92). ASMR in 2023 was highest in Oceania (18.4 per 100,000 population) and lowest in High-income North America (2.93 per 100,000 population). East Asia has the highest number of deaths (390,366) and Oceania has the lowest (1424). ASMRs all declined to varying degrees, with the largest decline in the East Asia region (EAPC = −3.81,95% CI: −4.07, −3.55). The highest ASDR in 2023 is in Oceania (472.87 per 100,000 people) and the lowest is in High-income North America (71.8 per 100,000 people). The region with the highest number of DALYs is East Asia (9139,067) and the lowest is Australasia (43,046). ASDR declined in Central Asia, Central Europe, East Asia, Eastern Europe, High-income Asia Pacific, High-income North America, and Western Europe. The Eastern Europe region (EAPC = −2.64,95% CI: −2.74, −2.54) had the largest decrease.

### 3.3. National burden of GC

**Figure 4.**
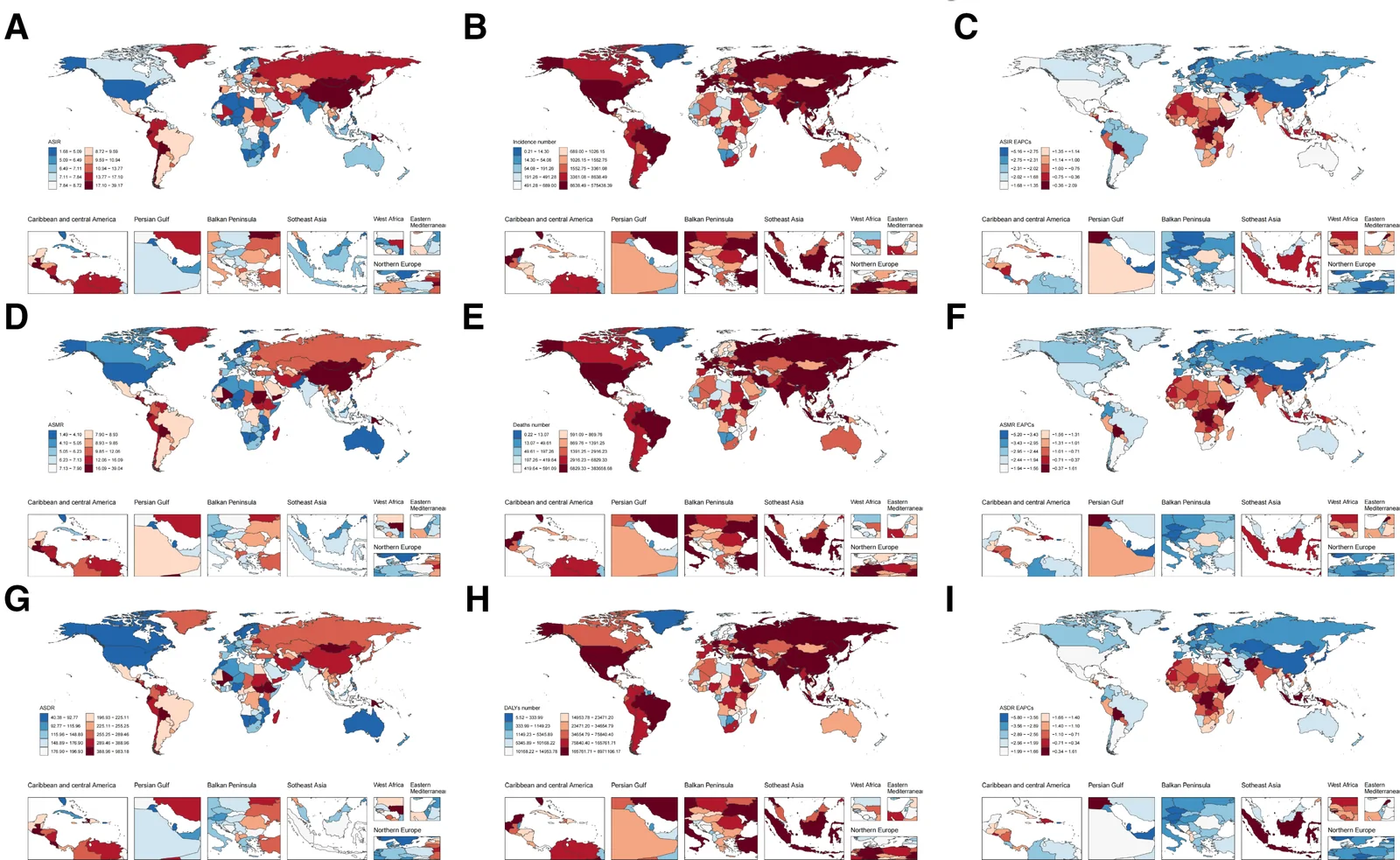
Global map. (A–C) ASIR, number of incidences, EAPC; (D–F) ASMR, number of deaths, EAPC; (G–I) ASDR, number of DALYs, EAPC. AAPC = average annual percentage change, ASIR = age-standardized incidence rate, ASMR = age-standardized mortality rate, ASDR = age-standardized DALY rate, DALY = disability-adjusted life year, EAPC = estimated annual percentage change.

In 2023, Mongolia (38.78 per 100,000), Plurinational State of Bolivia (35.39 per 100,000), and Solomon Islands (27.59 per 100,000) have the highest ASIRs of all countries. China (569,741), Japan (106,838) had the highest number of incidences, and United Arab Emirates (EAPC = −5.11 [−5.51, −4.70]) had the largest decrease in ASIR. Mongolia (38.66 per 100,000), Plurinational State of Bolivia (35.03 per 100,000) and Nauru (25.9 per 100,000) had the highest ASMR among all countries. China (379,761), India (86,039) and Japan (61,196) had the highest number of deaths, and Lebanon Republic (EAPC = 4..94 [4.49,5.39]) had the largest increase in ASMR. Mongolia (973.44 per 100,000), Plurinational State of Bolivia (831.36 per 100,000), and Republic of Guatemala (604.93 per 100,000) had the top 3 ASDRs for all countries. The top 3 countries in DALY numbers were Arab Republic of Egypt (22,528,492), Kyrgyz Republic (8882,283) and State of Eritrea (2422,360). The country with the largest decrease in ASDR was Republic of Korea (EAPC = −5.74 [−5.95−5.53]). (Figure 4, Table S3, S4, Supplemental Digital Content 3, 4).

### 3.4. Trends in SDI and GC burden

**Figure 5.**
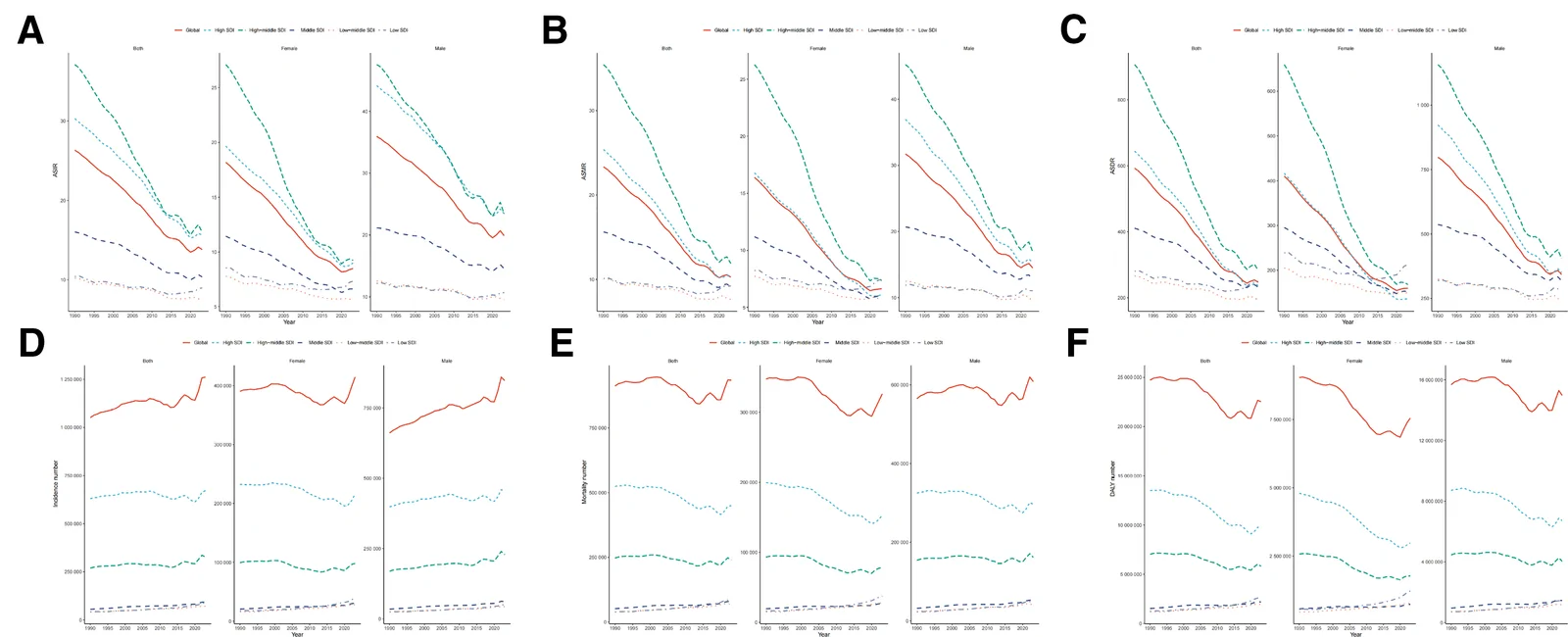
Area-Time Trend Analysis. (A–C) ASIR, ASMR, ASDR; (D–F) Number of incidence, number of deaths, number of DALYs. ASIR = age-standardized incidence rate, ASMR = age-standardized mortality rate, ASDR = age-standardized DALY rate, DALY = disability-adjusted life year.

**Figure 6.**
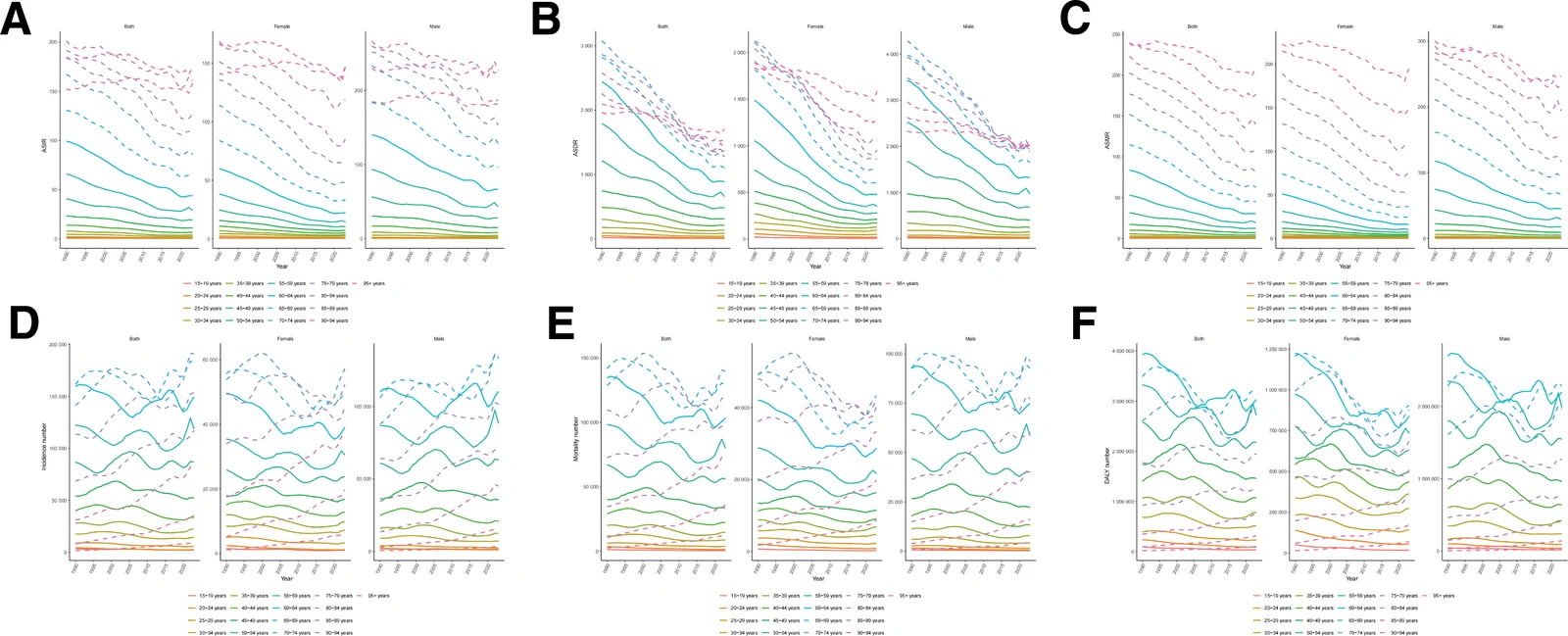
Age-time trend analysis. (A–C) ASIR, ASMR, ASDR; (D–F) Number of incidence, number of deaths, number of DALYs. ASIR = age-standardized incidence rate, ASMR = age-standardized mortality rate, ASDR = age-standardized DALY rate, DALY = disability-adjusted life year

The GBD database divides the world into 5 regions based on the quintiles of SDI, which are High SDI region, High-middle SDI region, Middle SDI region, Low-middle SDI region and Low SDI region. From 1990 to 2023, the ASIR, ASMR, and ASDR in the 5 SDI regions showed a significant decreasing trend, respectively. There were no significant gender differences in the trends of the number of incidence, mortality, and DALY (Figure 5–6, Table 1 and S1, Supplemental Digital Content 1). Except for High SDI and High-middle SDI regions, all indicators in the remaining SDI regions were lower than the global level. Among them, ASIR, ASMR, and ASDR were the highest in the High-middle SDI region (15.99, 11.88, and 282.26 per 100,000, respectively), and their decreases were also the greatest (EAPC −2.84, −3.62, and −3.88, respectively, *P* < .05). The High SDI region had the highest number of incidences, deaths, and DALYs (671,662, 449,993, and 9,708,202, respectively).

**Figure 7.**
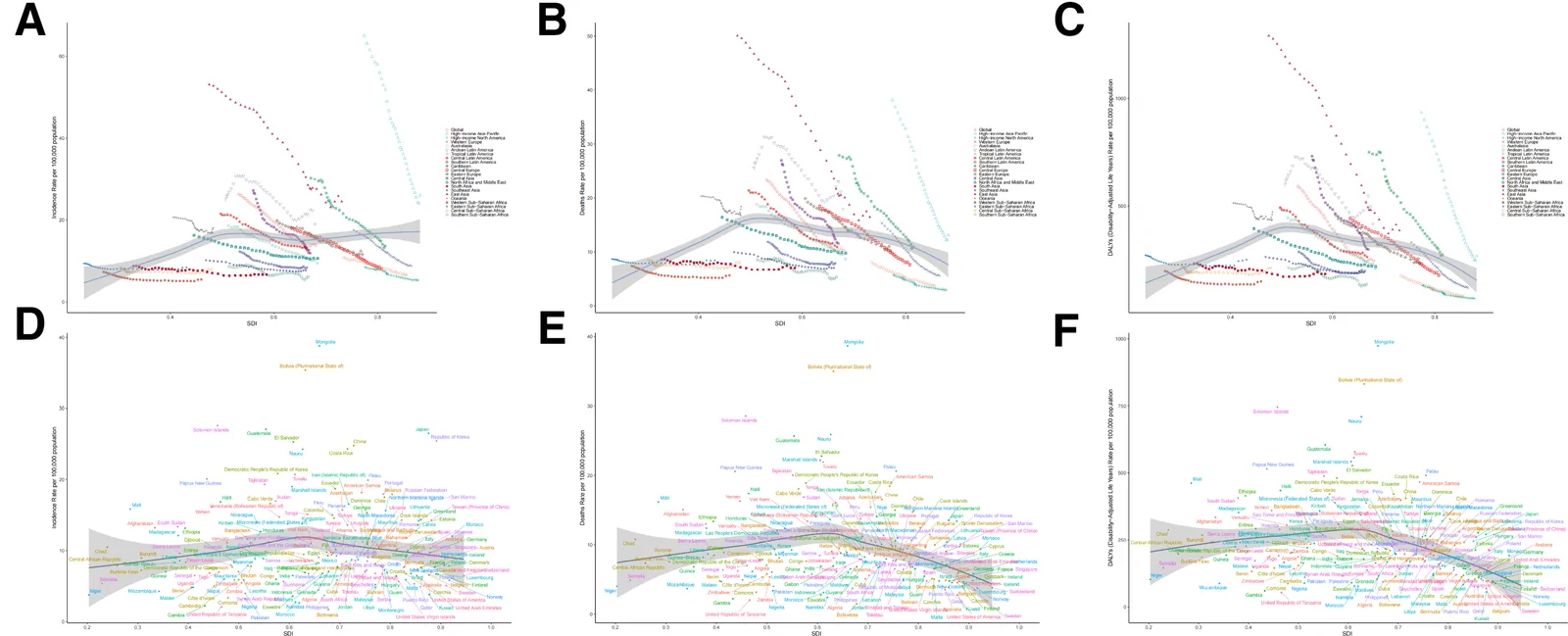
GC Burden and SDI Trend Analysis. (A–C) SDI analysis of ASIR, ASMR, ASDR for 21 regions; (D–F) SDI analysis of ASIR, ASMR, ASDR for 204 countries. ASIR = age-standardized incidence rate, ASMR = age-standardized mortality rate, ASDR = age-standardized DALY rate, SDI = socio-demographic index.

We further analyzed the association between GC burden and SDI over the last 3 decades (Fig. 7). Across the 21 GBD regions, ASIR generally increased with SDI, whereas ASMR and ASDR showed an increasing-then-decreasing pattern with increasing SDI.

### 3.5. Decomposition analysis

**Figure 8.**
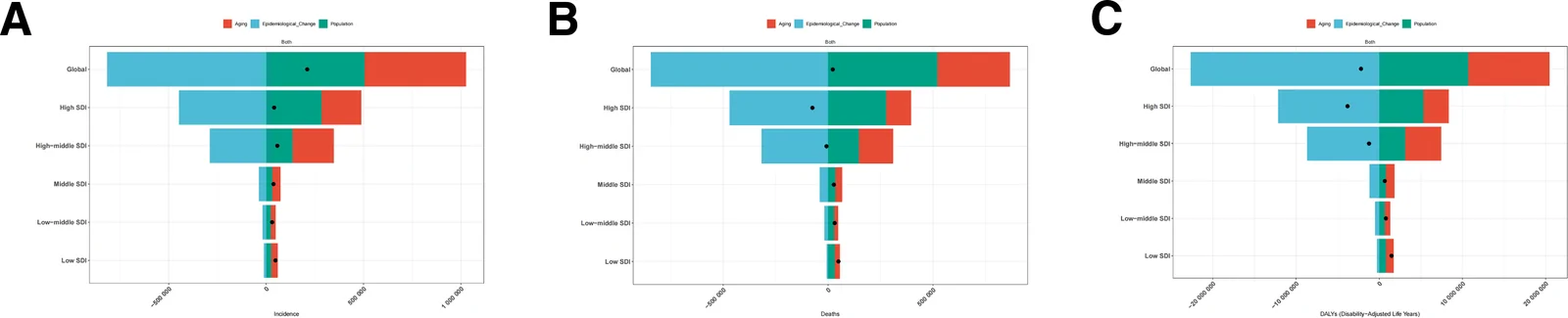
Decomposition analysis. (A) ASIR; (B) ASMR; (C) ASDR. ASIR = age-standardized incidence rate, ASMR = age-standardized mortality rate, ASDR = age-standardized DALY rate.

Figure 8 shows the decomposition analysis of global aging, demographic and epidemiologic change factors in the 5 SDI regions. The decomposition analysis shows that 1035.7% of DALY changes are associated with epidemiologic changes, followed by population (−489.88%) and aging (−445.82%). Among the different SDI regions, the region most affected by the 3 population-level determinants was located in the High-middle SDI region with 710.25% of epidemiologic change, −254.42% of population and −355.83% of aging. The Low-middle SDI region was the least affected by epidemiologic change (−63.1%), and the Low SDI region was the least affected by population (53.33 %), and aging (3.49%). From this point of view, the contribution of these 3 determinants to the relevant indicators varies considerably. The contribution of aging and population to DALY is positive in the High-middle SDI and High SDI regions, while the epidemiologic trend is opposite. The reverse is true for the Remaining SDI region and these 2 regions. Other noteworthy points are that the contribution of epidemiologic change (−13599.61 %), population (8410.62 %), and aging (5288.99 %) to the overall DALY change were all largest in Southern Latin America. Among the different regions, those least affected by epidemiologic changes, population and aging were Oceania (−6.18 %), Central Sub-Saharan Africa (43.29 %) and Western Europe (−8.47 %), respectively.

### 3.6. Health inequality analysis

**Figure 9.**
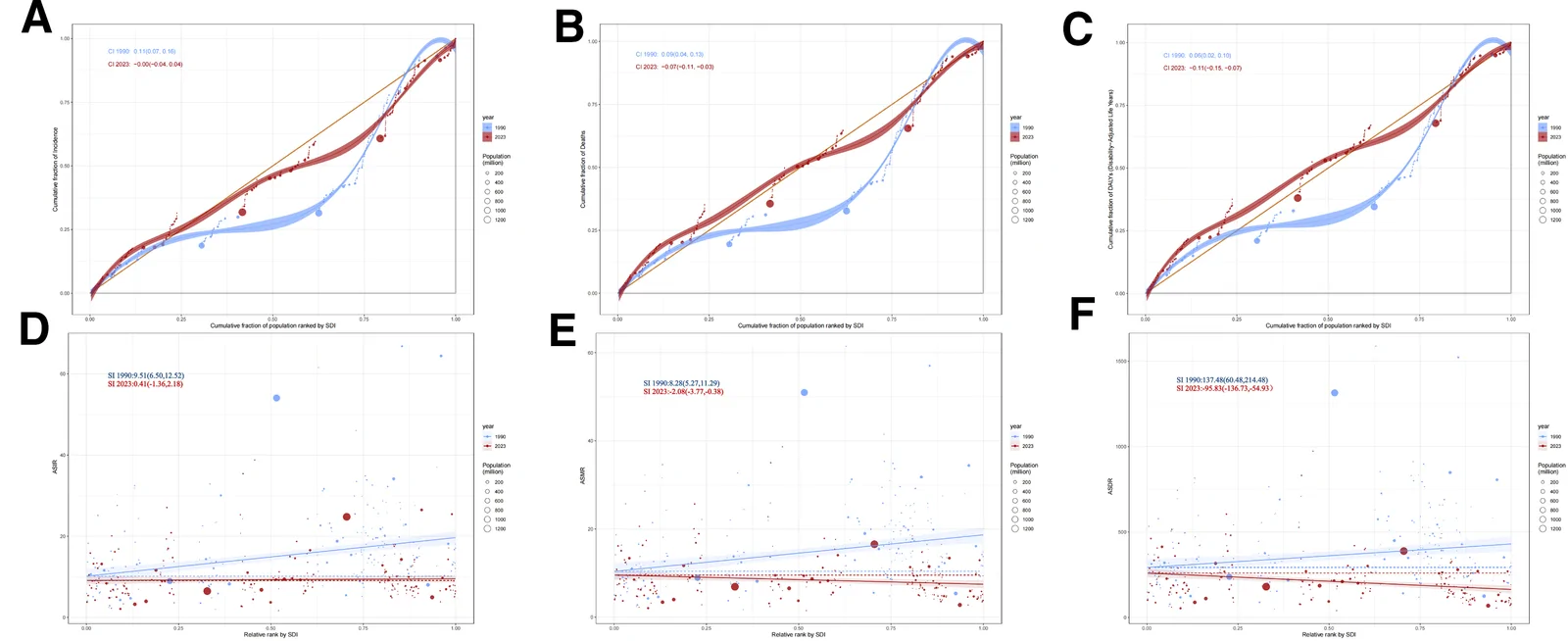
Analysis of health inequalities. (A–D) ASIR; (B–E) ASMR; (C–F) ASDR. ASIR = age-standardized incidence rate, ASMR = age-standardized mortality rate, ASDR = age-standardized DALY rate.

In terms of GC burden, the results of the health inequality analysis showed absolute and relative inequalities associated with SDI (Fig. 9).The results of the slope index slope index of inequality showed that incidence rate ranged from 9.51 (95% CI: 6.50–12.52) in 1990 to 0.41 (95% CI: −1.36, 2.18) in 2023. Mortality rates from −8.28 (95% CI: −11.29, −5.27) in 1990 to −2.08 (95% CI: −3.77, −0.38) in 2023. DALY rate from 137.48 (95% CI: 60.48–214.48) in 1990 to −95.83 (95% CI: −136.73, −54.93) in 2023. The above results suggest that morbidity shows a greater disease burden in areas with high SDI, mortality shows a greater disease burden in areas with low SDI, and the greater disease burden of DALY shifts from areas with high SDI to areas with low SDI, but overall the absolute inequality of the GC burden improves.The results of the centralized index CI show that the incidence (0.11 vs 0.00) and mortality (0.09 vs −0.07) in 2023 suggest that the relative inequality of the GC burden has improved, and that the DALY in 2023 (0.06 vs −0.11) demonstrates that low SDI areas are beginning to bear a greater share of the GC burden, and that the gap is widening further.

### 3.7. Frontier analysis

**Figure 10.**
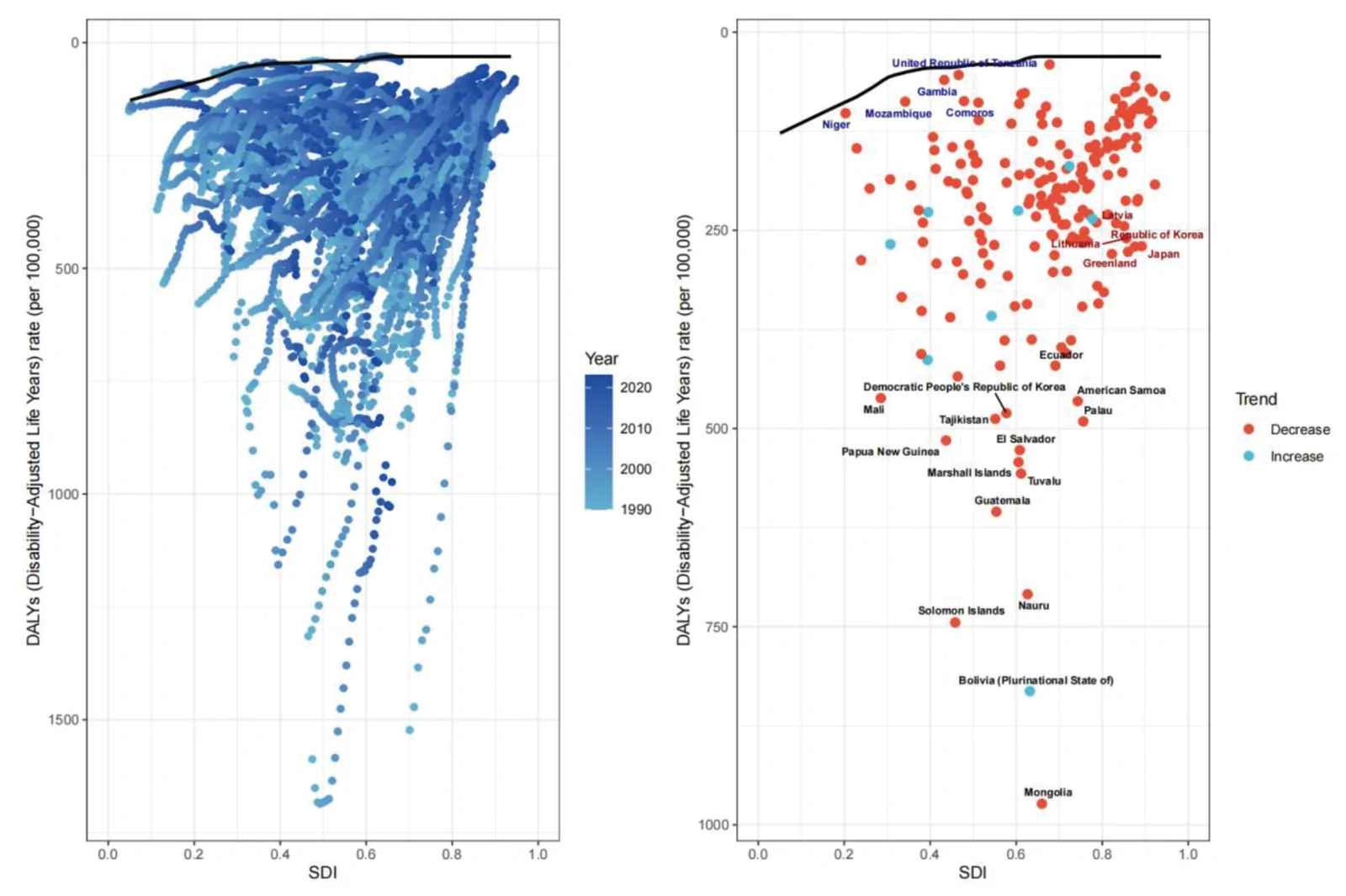
Frontier analysis.

This study utilized GC data from the GBD database for 1990 to 2023 to conduct a frontier analysis based on ASDR and SDI, combined with national and regional levels of development, to explore potential room for improvement in GC-related epidemiological indicators (Figure 10, Table S5, Supplemental Digital Content 5). Borderlines indicate regions with the lowest age-standardized DALY (best performer) according to the corresponding SDI. The effective distance between a country and the borderline is defined as the gap between a country’s observed and potential age-standardized DALY. Figure 10 shows the frontier analysis of trends in DALY. The 15 countries and territories with the largest effective differences were Mongolia, Bolivia (Plurinational State of), Solomon Islands, Nauru, Guatemala, Tuvalu, Marshall Islands, El Salvador, Papua New Guinea, Palau, Tajikistan, the Democratic People’s Republic of Korea, American Samoa, Mali, and Ecuador, with effective differences ranging from 389.96 to 942.67. Frontier analyses reveal potential room for improvement in reducing the burden of GC across countries and regions, with effective differences generally increasing somewhat as socio-demographics evolve, suggesting that countries or regions with higher SDIs have greater potential for burden improvement.

### 3.8. Age-period-cohort (APC) modeling

The age-specific effect is a widely used concept in epidemiology and social sciences that refers to the influence of age in a particular phenomenon (e.g., disease incidence, mortality, behavioral practices, etc). This effect is usually understood by analyzing the health status of people of different ages, their behavioral patterns, and the risk factors they face for specific outcomes.

**Figure 11.**
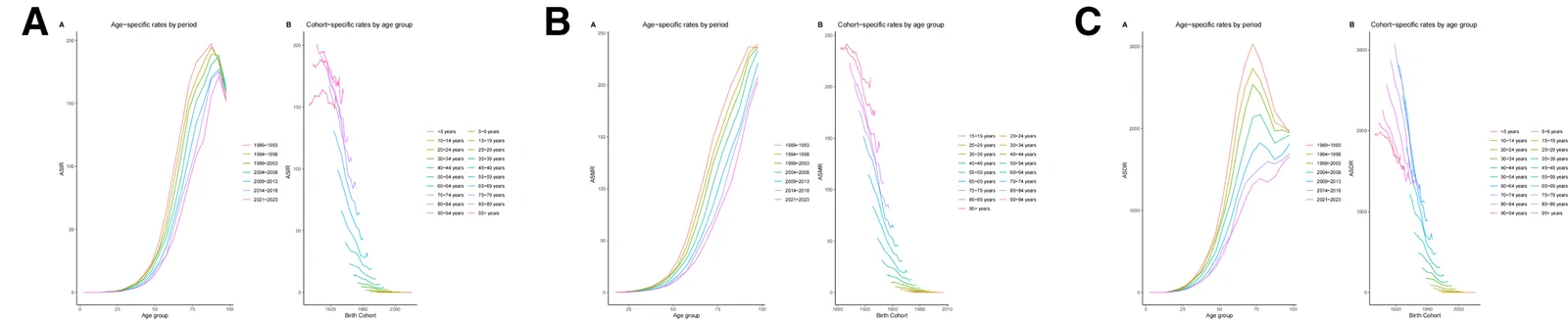
Age-specific effects. (A) ASIR; (B) ASMR; (C) ASDR. ASIR = age-standardized incidence rate, ASMR = age-standardized mortality rate, ASDR = age-standardized DALY rate.

We analyzed the effect of age, period, and cohort on the burden of GC by dividing the ASIR, ASMR, and ASDR and demographic data for GC into 7 consecutive time periods ranging from 1989 to 1993 (median, 1991) to 2019 to 2023 (median, 2021), and 20 consecutive cohorts with 5-year intervals (Fig. 11). Between 1989 and 2023, ASIR for GC increased and then decreased with age, while ASMR showed an increase. ASDR first increased and then decreased with age between 1989–1993 and 2009–2013, and showed a constant increase after 2014–2018. ASIR, ASMR, and ASDR all declined over time. ASDR birth cohort data showed that ASIR, ASMR, and ASDR all generally exhibited a declining trend.

**Figure 12.**
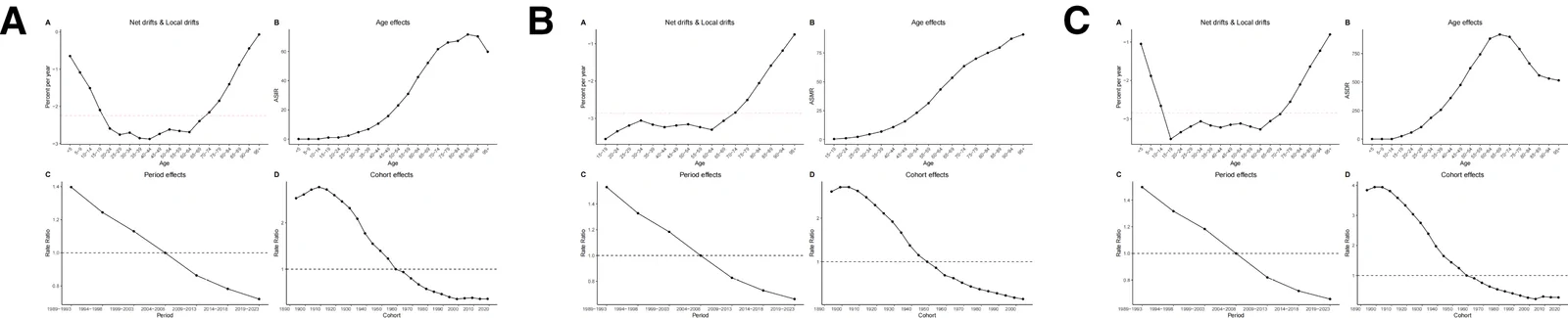
APC model. (A) ASIR; (B) ASMR; (C) ASDR. ASIR = age-standardized incidence rate, ASMR = age-standardized mortality rate, ASDR = age-standardized DALY rate.

Net drift represents the overall annual percentage change over the entire study period, whereas localized drift represents the annual percentage change for each age group relative to the net drift (Fig. 12). The overall net drift of ASIR, ASMR, and ASDR for GC was −2.248 % (95% CI: −7.119–2.880), −2.865 % (95% CI: −2.952--2.778), and −2.847 % (95% CI: −24.781–25.482), respectively. This reflects a decrease in ASMR to some extent during the study period, with ASIR and ASDR decreasing and then increasing. In males, ASIR (−2.113% (95% CI: −6.794–2.802) vs −2.558% (95% CI: −5.448–0.420)), ASMR (−2.789% (95% CI: −2.903–−2.674) vs −3.105% (95% CI: −3.188–3.022) respectively), ASDR (-2.780% (95% CI: −23.404–23.349) vs −3.029% (95% CI: −18.204–14.961), respectively) declined less than women.

Localized drift reflects additional age-related changes in trends associated with epidemiologic indicators. For GC, ASIR and ASDR showed an increasing and then decreasing trend with age, and ASMR showed an increasing trend. Period effects showed a decreasing trend in ASIR, ASMR, and ASDR over time throughout the study period.Cohort effects showed a decreasing trend in all 3 values, suggesting a decrease in the risk of developing GC in the recent birth cohort.

### 3.9. 2035 projections of the burden of GC: a Bayesian age cohort model based analysis

**Figure 13.**
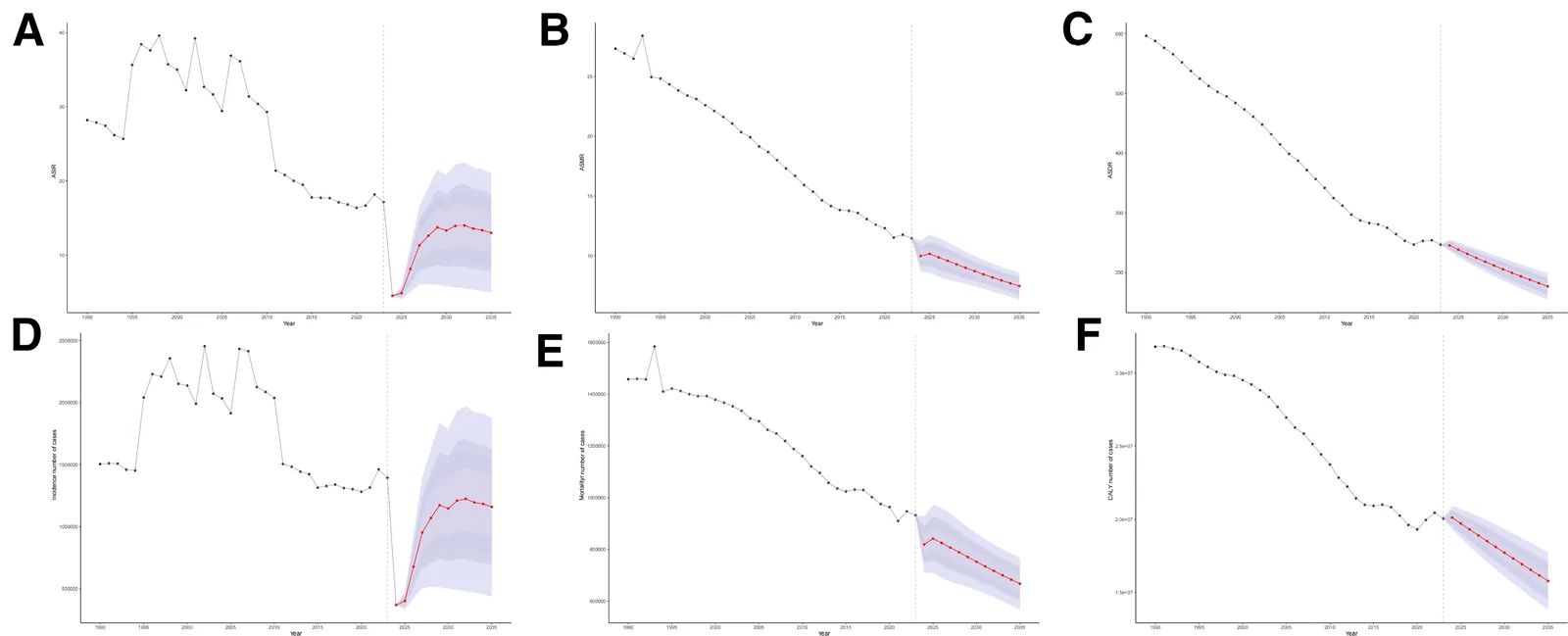
BAPC model. (A, D) ASIR and number of incidences; (B, E) ASMR and number of deaths; (C, F) ASDR and number of DALYs.

**Figure 14.**
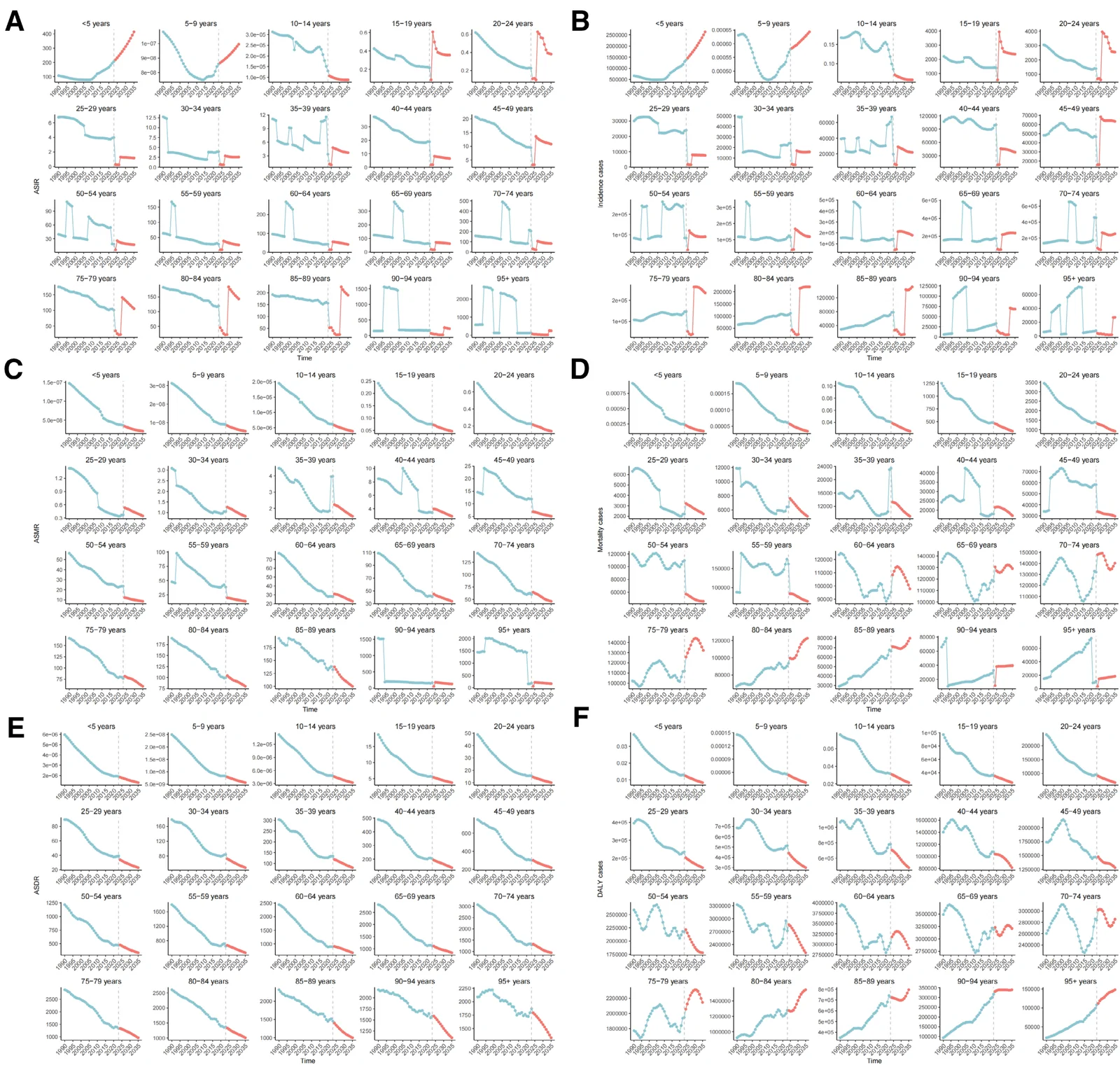
BAPC model based on age subgroups. (A, B) ASIR and number of incidences; (C, D) ASMR and number of deaths; (E, F) ASDR and number of DALYs. ASIR = age-standardized incidence rate, ASMR = age-standardized mortality rate, ASDR = age-standardized DALY rate, BAPC = Bayesian age-period-cohort, DALY = disability-adjusted life year, SDI = socio-demographic index.

The BAPC analysis projected the global burden of GC in 2035 and subgrouped it in terms of sex and age groups (Figs. 13–14).The number of GC incidence cases is projected to reach 1,159,745 in 2035, with more pronounced upward trends in the under-24 and over-75 age groups.The ASIR showed a decreasing trend, and the changes in the age groups converged with the changes in the number of incidence cases. The number of deaths is projected to decrease to 667,145, but with an increasing trend in the 75 + age group. ASMR is projected to decrease to 7 per 100,000, with the same decreasing trend across all age groups. DALY is projected to decrease to 15,783,072 and ASDR is projected to decrease to 177 per 100,000, with decreasing trends in the DALY count and ASDR. ASDR shows the same downward trend for all age groups. However, DALY counts show an increasing trend in the 75+ age group. There is no significant change in the male and female population.

## 4. Discussion

This research thoroughly evaluated the global trends in the burden of GC from 1990 to 2023, utilizing data from GBD 2023. Over this period, there was a 20.04% increase in the incidence of GC worldwide, with deaths rising to 934,795, while the number of DALYs exhibited an 8.85% decline since 1990. Nevertheless, the ASIR, ASMR, and ASDR for GC have generally trended downward since 1990. This trend underscores the success of public health initiatives, particularly in regions with high and medium-high SDI, where advancements in early detection and treatment of GC have markedly improved. For instance, while ASIR remains elevated in East Asia, the overall incidence has dropped due to enhanced public health policies.^[14–19]^ In high-income nations, the reduction in ASIR has been even more pronounced, closely tied to their sophisticated healthcare systems and proactive screening measures.^[20]^ Conversely, in low- and middle-income countries, the ASIR decline has been less substantial, with some areas experiencing an upward trend, indicating that significant obstacles in GC prevention and management still persist globally. A decrease in epidemiological metrics does not necessarily imply a reduced cancer burden in high-risk nations. For instance, factors such as an aging demographic, population growth, and unhealthy lifestyles could be contributing to the persistent rise in cancer incidence and mortality across various regions. Furthermore, the impact of noncommunicable diseases, including cardiovascular and chronic respiratory illnesses, continues to grow, particularly among older populations, resulting in increased mortality rates and diminished quality of life.^[21]^ These shifts not only influence the health outcomes of individuals but also exert pressure on healthcare systems and economic progress in these countries. The observed differences in GC burden across regions, sexes, and socioeconomic levels may reflect variations in population aging, demographic structure, healthcare accessibility, and exposure patterns. However, because this study is based on aggregated GBD estimates, causal relationships cannot be established. The increase in the absolute number of GC cases and deaths despite declining age-standardized rates may be partly related to demographic changes, particularly population expansion and aging. As older populations generally experience a higher risk of GC, demographic transitions may continue to influence the future global disease burden.

Globally, the impact of GC differs markedly across various SDI regions. The ASIR, ASMR, ASDR, along with morbidity counts, mortality figures, and DALYs are notably greater in High SDI and Upper-middle SDI regions compared to global averages. This finding is intricately linked to the level of medical resources, the support provided by policies, available technologies, and the extent of public health awareness in individual regions and nations. During the 1980s, high SDI countries exhibited a consumption rate of processed meat products at 56.3 kg per person annually, which was 3.2 times the rate seen in low SDI nations. Long-term exposure to elevated nitrate levels has contributed to the emergence of gastric mucosal metaplasia, leading to a significant increase in both the morbidity and mortality associated with GC.^[22,23]^ However, following the introduction of food safety policies, such as the Guidelines for Food Salt Restriction in Japan in 1995, the consumption of preserved foods in the high SDI regions diminished by 61% from 1990 to 2020, demonstrating a rapid effect of risk factor management.^[24]^ Furthermore, the early detection rate of GC in high SDI regions stands at 54.3%, which is 4.3 times greater than the 12.7% rate observed in low SDI regions. Additionally, the widespread adoption of endoscopic mucosal dissection has elevated the 5-year survival rate to 82.4%, whereas surgical accessibility in low SDI regions remains below 30%.^[5,25]^ This trend in the evolution of disease burden corroborates the concept of “epidemiologic transition theory” within high SDI regions, shifting GC from being classified as a “disease of poverty” to one that is “preventable and manageable” through comprehensive prevention and control strategies.

In comparison to women, men experience a more significant impact from GC. The ASIR, ASMR, and ASDR are approximately double in men compared to women, potentially influenced by a range of factors, including physiological, genetic, and lifestyle differences. The levels of androgen receptor expression are 3.8 times greater in the gastric mucosa of men than in women. This receptor facilitates intestinal epithelial chemotaxis by activating the Wnt/β-catenin pathway, while estrogen in women provides a protective role by inhibiting the IL-6/STAT3 signaling cascade.^[23]^ Research has shown that the number of aberrant methylation sites in the DNA of male GC tissues is 1.9 times greater than that found in females. Notably, the hypermethylation rate in the promoter region of the CDKN2A gene is 58.3% for males, significantly exceeding the 32.7% observed in females.^[26]^ Beyond biological reasons, variations in risk-related behaviors play a crucial role: globally, smoking prevalence is 5.6 times higher among men (34.7%) than women (6.2%). Compounds such as NNK [4-(methylnitrosoamino)-3-pyridinyl-1-butanone], generated from tobacco, can accelerate cancer development and progression through mechanisms such as oxidative stress, inflammation induction, and disruption of the mucus bicarbonate barrier. The likelihood of cancer among smokers rises with both the amount smoked daily and the duration of smoking. Specifically, the risk of tumors due to smoking is 1.67 times higher compared to nonsmokers, and the chance of developing intestinal epithelial hyperplasia in smokers is 3 times that of nonsmokers.^[27]^ Additionally, occupational hazards, including those in mining and metal-processing sectors, increase men’s exposure to carcinogens like polycyclic aromatic hydrocarbons by a factor of 3.2.^[23,26]^ The higher GC burden observed among males may be associated with differences in demographic characteristics, exposure patterns, and healthcare-related factors. However, because this study used aggregated population level estimates, these factors should be interpreted as possible related factors rather than established causal mechanisms.

Moreover, the impact of GC is considerably more pronounced in older adults. The rate of GC diagnosis increases with advancing age for both genders. Firstly, the cumulative exposure to carcinogenic agents is considerably more significant in the elderly demographic. Research indicates that prolonged infection with Helicobacter pylori (lasting 10 years or more) can heighten the risk of developing GC by a factor of 4.7. Notably, the average infection duration among individuals aged over 60 years is 28.3 years, which is substantially longer than that observed in younger individuals.^[22,25]^ Secondly, age-related alterations in the gastric mucosa hasten the cancer development process: in older patients, the length of telomeres in gastric mucosal tissues is reduced by 38% compared to younger counterparts, resulting in increased genomic instability. Concurrently, the rate of DNA methylation abnormalities is 2.9 times greater.^[5,23]^ Furthermore, the responses to treatments and the patterns of drug resistance in older patients could diverge significantly from those seen in younger patients, highlighting the necessity for a tailored therapeutic approach for the elderly.^[28]^

According to the BAPC model, it is projected that by 2035, the number of cases among individuals aged over 45 will continue to rise, whereas the incidence in younger demographics is expected to decline. This indicates the necessity for proactive planning and the implementation of effective strategies to mitigate this trend. Specifically, there is an urgent need to enhance health management for the middle-aged and elderly population, while also focusing on health education for younger individuals to meet long-term goals of disease prevention and control.

The strength of this paper is rooted in the utilization of the most recent data from the GBD database, which offers an extensive time-series analysis and integrates various statistical models to forecast future trends. Nonetheless. Several limitations should be acknowledged. First, GBD estimates are generated using modeling approaches and may differ from directly observed cancer registry data. Second, differences in data availability and quality among countries may influence international comparisons. Third, BAPC projections assume continuation of historical trends and may not fully capture future changes in prevention strategies or healthcare development. Finally, individual-level risk factors and causal pathways cannot be evaluated using aggregated population level data. Additional limitations include that GBD estimates are model based rather than direct registry data; differences in data quality among countries may affect comparisons; BAPC projections assume continuation of historical trends; and individual-level risk factors cannot be evaluated.

In conclusion, the global burden of GC remains high, and early screening and prevention can effectively improve the situation through active public health strategies and multifaceted interventions. Future studies should continue to explore the diverse factors affecting the development of GC in order to promote more accurate prevention and treatment measures.

## 5. Conclusion

The global disease burden of GC has increased significantly over the past 3 decades, especially in terms of the number of deaths. The burden of GC is higher in male patients than in females. In the face of this increasing trend, countries need to develop targeted prevention and control strategies, especially in countries and regions with increasing aging and low SDI levels. Although the absolute burden remains substantial, age-standardized rates have generally declined. Future strategies should address population aging, demographic change, and socioeconomic disparities. Future research should continue to monitor and evaluate the effectiveness of these strategies and explore more innovative ways to reduce the disease burden of GC.

## Acknowledgments

We sincerely appreciate the significant contributions made by all the authors towards this study, their invaluable efforts have been instrumental in its success.

## Author contributions

**Conceptualization:** Liying Ma, Juan Ou, Zijun Qiu, Xi Lin, Dingyu Guo, Bing Yan.

**Data curation:** Liying Ma, Juan Ou, Zijun Qiu, Xi Lin, Dingyu Guo, Bing Yan.

**Formal analysis:** Liying Ma, Juan Ou, Zijun Qiu, Xi Lin, Dingyu Guo, Bing Yan.

**Funding acquisition:** Liying Ma, Juan Ou, Xi Lin, Dingyu Guo, Bing Yan

**Investigation:** Liying Ma, Bing Yan.

**Writing – original draft:** Bing Yan.

**Writing – review & editing:** Bing Yan.










